# Adult health and transition stage-specific rotenone-mediated *Drosophila* model of Parkinson’s disease: Impact on late-onset neurodegenerative disease models

**DOI:** 10.3389/fnmol.2022.896183

**Published:** 2022-08-09

**Authors:** Mohamad Ayajuddin, Limamanen Phom, Zevelou Koza, Priyanka Modi, Abhik Das, Rahul Chaurasia, Abuno Thepa, Nukshimenla Jamir, Kelevikho Neikha, Sarat Chandra Yenisetti

**Affiliations:** Drosophila Neurobiology Laboratory, Department of Zoology, Nagaland University (Central), Lumami, India

**Keywords:** *Drosophila*, Parkinson’s disease, rotenone, health phase, transition phase, dopamine

## Abstract

Parkinson’s disease (PD) affects almost 1% of the population worldwide over the age of 50 years. Exposure to environmental toxins like paraquat and rotenone is a risk factor for sporadic PD which constitutes 95% of total cases. Herbicide rotenone has been shown to cause Parkinsonian symptoms in multiple animal models. *Drosophila* is an excellent model organism for studying neurodegenerative diseases (NDD) including PD. The aging process is characterized by differential expression of genes during different life stages. Hence it is necessary to develop life-stage-matched animal models for late-onset human disease(s) such as PD. Such animal models are critical for understanding the pathophysiology of age-related disease progression and important to understand if a genotropic drug/nutraceutical can be effective during late stages. With this idea, we developed an adult life stage-specific (health and transition phase, during which late-onset NDDs such as PD sets in) rotenone-mediated *Drosophila* model of idiopathic PD. *Drosophila* is susceptible to rotenone in dose-time dependent manner. Rotenone-mediated fly model of sporadic PD exhibits mobility defects (independent of mortality), inhibited mitochondrial complex I activity, dopaminergic (DAergic) neuronal dysfunction (no loss of DAergic neuronal number; however, reduction in rate-limiting enzyme tyrosine hydroxylase (TH) synthesis), and alteration in levels of dopamine (DA) and its metabolites; 3,4-Dihydroxyphenylacetic acid (DOPAC) and Homovanilic acid (HVA) in brain-specific fashion. These PD-linked behaviors and brain-specific phenotypes denote the robustness of the present fly model of PD. This novel model will be of great help to decipher life stage-specific genetic targets of small molecule mediated DAergic neuroprotection; understanding of which is critical for formulating therapeutic strategies for PD.

## Introduction

Parkinson’s disease (PD) is a neurodegenerative disease (NDD) affecting the movement in the aging human population (Tysnes and Storstein, 2017) and the second most common NDD affecting about 1% of the population over 50 years (Ayajuddin et al., 2018), 1.5–2% over the age of 60 years, and 4% of the elderly individuals over the age of 80 years (Marino et al., 2020). More than 6 million individuals across the globe are suffering from PD (Armstrong and Okun, 2020). A central pathological hallmark of PD is the dopaminergic (DAergic) neuronal degeneration in the S*ubstantia Nigra pars compacta* (SNpc) leading to pathological features such as tremors, stiffness, slowness, and psychological or cognitive problems such as cognitive decline, depression, and anxiety (Armstrong and Okun, 2020). Multiple non-motor symptoms such as sleep disturbances, bladder problems, constipation, fatigue, loss of energy, hypotension, and sexual problems are also involved in PD which hamper the quality of life (Pfeiffer, 2017).

Multiple environmental toxins have been implicated to provoke neurotoxicity in animal PD models. Among those, the compound rotenone has been broadly used as a neurotoxin in experimental animal models for PD. Being a lipophilic molecule, rotenone can cross the blood-brain barrier (Fuzzati-Armentero et al., 2019). It is a systemic mitochondrial complex-I inhibitor (Mariano and Mónica, 2019) that impairs oxidative phosphorylation of the electron transport chain (ETC), thereby producing reactive oxygen species and inducing cytotoxicity and apoptosis (Wang et al., 2021). The rotenone model is very attractive and relevant since it replicates the crucial pathological and biochemical features of PD including oxidative stress, mitochondrial dysfunction, and DAergic degeneration (Sherer et al., 2003; Miyazaki et al., 2020).

Developing an animal model is critical to understand the pathophysiology of an NDD such as PD. Among animal model systems, *Drosophila melanogaster* has been paid more attention because of its simpler morphology, simpler nervous system, and highly conserved disease-causing genes with humans. Comparative analysis of whole-genome sequencing revealed that 77% of human disease-causing genes have fly homologs/orthologues (Reiter et al., 2001; Tello et al., 2022) and 40% of overall similarity at nucleotide or protein levels with 80–90% or higher in the conserved domain (Pandey and Nichols, 2011), which emphasizes the utility of “little fly” to understand and address human disease.

Prevalently, PD is a late-onset NDD. However, several researchers developed *Drosophila* PD model using young (5–10 days) animals (Coulom and Birman, 2004; Cassar et al., 2015; Pandareesh et al., 2016; Nguyen et al., 2018). Studies regarding susceptibility genes and pathways of PD under neurotoxin treatment have also been conducted mostly using flies belonging to the adult health phase (up to 20 days old) (Cassar et al., 2015; Sur et al., 2018; Maitra et al., 2021).

The aging process is characterized by the differential expression of multiple genes during different life stages (Pletcher et al., 2002; Bordet et al., 2021). The adult life of *Drosophila* is categorized into health (no natural death occurs), transition (slight decline in the mortality curve showing 10% death), and senescence stage (steady decline in mortality curve represented by the window between the end of the transition phase till maximum life span of the fly) (Arking et al., 2002). Analysis of these adult life stages in model organisms has revealed that they are characterized by different patterns of gene expression (Pletcher et al., 2002; Arking, 2009, 2015), which is similar to that of the equivalent life stages of humans. A transcriptomic analysis using microarrays to compare the gene expression profiles of different life stages of *Drosophila melanogaster* has identified 1,184 genes with pronounced differences in expression level between young and old age groups (Bordet et al., 2021). The life stages of *Drosophila* are believed to have both common and unique complex processes and can be influenced independently by a relatively large number of stage-associated pathways (Soh et al., 2013). Our laboratory has previously demonstrated in the *Drosophila* PD model that nutraceutical curcumin’s DAergic neuroprotective efficacy is adult life stage-specific (Phom et al., 2014), which further signifies the importance and necessity of developing life stage-specific animal models for late-onset NDDs such as PD.

Here, we made an effort to develop neurotoxin rotenone (which inhibits mitochondrial complex I of ETC) mediated life stage-specific *Drosophila* model of sporadic PD. Adult *Drosophila* life stages constitute (a) health phase [5 days (early health phase); 30 days old (late health phase)] and (b) transition phase (50 days old; late-onset NDDs set in humans during transition phase). This model will be a good tool to screen potential therapeutic compounds and identify their molecular targets of activity, knowledge of which will assist to develop novel therapeutic strategies for PD.

## Material and methods

### Fly husbandry

Oregon K (OK; procured from National *Drosophila* Stock Center, Mysuru University, Mysuru, India) male flies of *D. melanogaster* were used in the present study. The flies were raised at 22 ± 1°C with 12 h (Hrs) light and dark cycle in a fly incubator (Percival, United States). The flies were fed with a culture medium composed of sucrose, yeast, agar-agar, and propionic acid (Phom et al., 2014). For collecting the flies, they were mildly anesthetized with a few drops of diethyl ether. Only 25 flies were kept in each vial containing fresh media. The collected flies were transferred to a fresh media vial every 3rd day. The 4 to 5-day flies were used for further experiment while late health span and transition phase flies were kept transferring routinely for every 3rd day till they reached a specific life stage and then were used for experiments.

### Chemicals

The required chemicals viz., Rotenone (Sigma, Cat: R8875), Curcumin (Sigma, Cat: 1386) and DMSO (Sigma, Cat: D8418) were used for feeding procedures. Standard dopamine (DA; Sigma-Aldrich, Cat: H8502) and its metabolites 3,4-Dihydroxyphenylacetic acid (DOPAC; Sigma-Aldrich, Cat: 11569) and Homovanilic acid (HVA; Sigma-Aldrich, Cat: 69673) were used for quantifying DA and metabolites. Phosphate-buffered Saline (PBS; HiMedia, India, Cat: ML023), Potassium ferricyanide (KCN; HiMedia, India, Cat: GRM627), and Nicotinamide adenine dinucleotide (NADH; SRL. India, Cat: 44018) were used for quantifying mitochondrial complex I activity. Paraformaldehyde (Sigma, Cat: I58127), Triton X-100 (Sigma, Cat: T8787), Normal Goat Serum (NGS; Vector Lab, Cat: S1000), VECTASHIELD mounting medium (Vector Labs, CA, United States, Cat: H1000), Rabbit anti-Tyrosine hydroxylase (anti-TH) polyclonal primary antibody (Millipore, MA, United States, Cat: Ab152), and Goat anti-rabbit IgG H&L (TRITC labeled) polyclonal secondary antibody (Abcam, MA, United States, Cat: Ab6718) were used for immunostaining.

### Rotenone resistance assay

To evaluate the toxicity of rotenone in *Drosophila*, OK male flies of the same age groups were transferred to a vial containing Whatman filter paper soaked with multiple concentrations of rotenone (10, 25, 50, 100, 250, 500, and 1,000 μM) prepared in 5% sucrose solution (stock rotenone solution prepared in DMSO), whereas control flies remain in 5% sucrose solution only (containing the same amount of DMSO). About 25 flies were placed in a vial. To avoid desiccation, flies were switched to a fresh vial with freshly prepared solutions every 24 h. The survivability of the flies was noted every 24 h.

#### Curcumin co-feeding regime

The flies were fed with ROT alone for the ROT treatment group or a combination of ROT and 250 μM curcumin for the co-feeding group. The control flies remain in 5% sucrose only while the curcumin *per se* group was fed with 250 μM curcumin prepared in a 5% sucrose solution. Climbing ability was assayed on the 5th day in the case of the health span flies, while it was assayed on the 2nd day for the transition phase flies.

### Negative geotaxis assay

The upward mobility of the flies, also called negative geotaxis assay, was assessed as described by Botella et al. (2004). In short, a single fly aspirated out of the vial was released into a plastic tube and acclimatized for 2 min. After gently tapping the fly to the bottom of the tube, the distance it could climb in 12 s was recorded. The same fly was given three chances and a minimum of 12 flies were noted for each concentration.

### Mitochondrial complex I–III activity

Mitochondrial NADH-Cytochrome C reductase (complex I–III) was assayed following Navarro et al. (2002). In brief, the dissected head and bodies of the flies were homogenized in mitochondrial extraction buffer. For complex I–III activity, 60 μg of the isolated mitochondria were mixed with phosphate buffer (0.1 M, pH 7.4). To this sample mixed with buffer, NADH (0.2 mM) and KCN (1 mM) were added and mixed for 10 s. The reaction was initiated with the addition of cytochrome C (0.1 mM) and the absorbance was recorded at 550 nm for 5 min using NanoDrop 2000, Thermo Scientific, United States. The total reaction volume was 1 ml. The activity was expressed as nmol cytochrome C reduced/min/mg protein (MEC = 19.6 mM^–1^ cm^–1^).

### Immunostaining of whole *Drosophila* brain

Mounting of the whole fly brain for fluorescence microscopy (Carl Zeiss, Axio Imager M2, with ZEN 2012 SP2 software, Germany) was done with modifications from Bayersdorfer et al. (2010). In brief, the male OK flies were fixed in 4% paraformaldehyde (PFA) containing 0.5% Triton (TX)-100, at room temperature for 2 h, and then washed five times after every 15 min in phosphate-buffered saline with 0.1% TX-100 (PBST), at room temperature (RT). Blocking was done using 0.5% TX-100 and 5% NGS for 120 min at RT. Then, the brains were incubated in a 1:250 ratio of primary antibody (anti-TH) for 72 h at 4°C. The excess primary antibody was removed by washing the brains for 5 × 15 min in PBST. Brains were then incubated with a 1:250 dilution of secondary antibody (TRITC labeled) for 24 h at room temperature under dark conditions. After thorough washing for 5 × 15 min in PBST, brains were mounted in VECTASHIELD mounting medium, and image acquisition was done on the same day.

The idea to quantify the fluorescence intensity of fluorescently labeled secondary antibodies (using Carl Zeiss, ZEN 2012 SP2 software) was adapted from the protocol of Navarro et al. (2014), where they quantified the fluorescence intensity of GFP reporters. In brief, prepared/stained brains were viewed under a fluorescence microscope. A Rhodamine filter was used for scanning the image. For image acquisition at 40×, a red dot test for visibility of neurons was done for all the brains. Z-stack programming with constant intervals was performed. For image processing, on method column, image subset and maximum intensity projection (MIP) with X–Y Plane were created. From 3D images of Z-stack, PAL, PPL1, PPL2, PPM1/2, and PPM3 (PAL- Protocerebral anterior lateral; PPL- Protocerebral posterior lateral; PPM- Protocerebral posterior medial) brain regions were selected. The images were enlarged to see clear neurites and a line was drawn around the neuron using a draw spline contour from graphics tools and the intensity sum was created in.xml format. The procedure was repeated for all the neurons in different clusters. Care was taken to select the fly brains with the same orientation.

### Quantification of dopamine and its metabolites using high-performance liquid chromatography

Brain-specific dopamine and its metabolites were quantified using high-performance liquid chromatography (HPLC-Thermo Scientific, Dionex Ultimate 3000) with an electrochemical detector (ECD) as described in Ayajuddin et al. (2021). In brief, the brains were homogenized in ice-chilled PBS. The supernatant collected after centrifugation was mixed with 5% TCA in a ratio of 1:1. About 50 μl of the supernatant was kept aside for protein quantification before mixing with TCA; 20 μl of standard DA, HVA, and DOPAC and 50 μl of the sample were loaded onto HPLC for quantification. MD-TM from Thermo Scientific (Cat: 701332) was used as the mobile phase and MCM 15 cm × 4.6 mm, 5 μ C-18 packed column (Thermo Scientific, Cat: 70-0340) was used as a stationary phase for elution of the monoamines. Inside the primary ECD containing two cells, detection of the monoamines was done at a range of -175 mV to + 225 mV as reduction and oxidation potential, respectively. The third cell which is part of the Omnicell (secondary ECD module) was set at + 500 mV to reduce background noise. The data collection rate was set at 5 Hz. Chromatograms were analyzed using Chromaleon^®^ 7, Thermo Scientific, United States.

### Data analysis

Statistical analysis was performed and graphs were prepared using GraphPad Prism 5.0 software and expressed as the mean ± standard error of the mean (SEM). Statistical significance was determined using a two-tailed unpaired *t*-test for the data with two groups. For the data with more than two groups, a one-way analysis of variance (ANOVA) followed by Newman–Keuls Multiple Comparison Test was performed. *p*-value < 0.05 was considered significant.

## Results

### *Drosophila* is susceptible to rotenone in a time-dose-dependent manner

**FIGURE 1:**
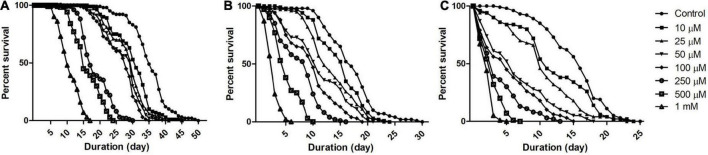
Dose and time-dependent mortality of *Drosophila* (OK) exposed to rotenone during different phases of adult life. Mortality pattern among adult male flies of early health span **(A)**, late health span **(B)**, and transition phase **(C)**, exposed to seven different concentrations of rotenone (10, 25, 50, 100, 250, 500, and 1,000 μM) showed concentration-dependent lethality. Mortality data were collected every 24 h for each group till all the flies were dead. There was no mortality up to the 6th day for health span flies and the 3rd day for late health and transition phase flies. Comparison of survival curves reveals that the response difference among different tested concentrations was significant (log-rank [Mantel–Cox] test. *p* < 0.0001).

To understand *Drosophila* susceptibility to rotenone, male OK flies of three life stages/phases (5 days: early health phase; 30 days: late health phase; and 50 days: transition phase) were exposed to multiple concentrations (10, 25, 50, 100, 250, 500, and 1,000 μM) of rotenone. The survivorship of flies was observed and recorded every 24 h. For the early health phase fly, at the exposed concentration of 500 μM rotenone, no mortality was observed up to the 7th day. For the late health and the transition phase fly, at the exposure of 25 and 10 μM of rotenone, respectively, no mortality was observed up to the 3rd day (Figure 1). Comparison of survival curves early health span (Figure 1A), late health span (Figure 1B), and transition phase (Figure 1C) showed that the response difference among all the tested concentrations was significant (Log-rank (Mantel-Cox) Test, *p* < 0.0001).

While modeling the PD-related pathology in animal systems including *Drosophila*, it is important to avoid the toxin concentration that kills the animal before the DAergic neurons degenerate. In such a situation, mechanistic insights the researcher generates using such animal models may not have relevance to the disease condition (Phom et al., 2014). Hence, it was determined to expose the flies belonging to the early health span phase to 500 μM rotenone, late health span phase to 25 μM rotenone, and transition phase to 10 μM rotenone and characterize the behavioral, biochemical, cytological, and molecular markers associated with PD at 5 days feeding window (in case of health span flies) and 2 days feeding window (in case of late health span and transition phase flies). At these time points, with these feeding protocols, no mortality was observed in all the stages of adult *Drosophila*. Hence, these models mimic the human PD condition (behavioral symptoms and DAergic neurodegeneration but no mortality).

### Rotenone induces locomotor defects in time and concentration-dependent manner

**FIGURE 2:**
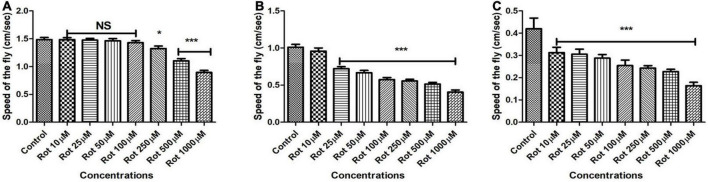
Assessing the climbing ability of *Drosophila* as determined by negative geotaxis assay after exposure to multiple concentrations of rotenone (10, 25, 50, 100, 250, 500, and 1,000 μM). About 500 μM of rotenone-induced clear mobility defects in the adult male fly of early health span on 5th day **(A)**, 25 μM rotenone in late health span fly on the 2nd day **(B)** and 10 μM rotenone in transition phase fly on the 2nd day **(C)** and there was no mortality at the selected time points. Observation of mere mobility defects but not mortality of fly is the reason behind selecting above mentioned toxin concentrations and durations of toxin exposure in adult life stage-specific fashion. Hence, these concentrations of toxin and window period of exposure were selected for further studies. Data were collected every 24 h for each group. One-way ANOVA followed by the Newman–Keuls Multiple Comparison Test showed a significant difference in mobility. *p < 0.05, ***p < 0.0001, NS, Not significant.

To establish the effect of rotenone on *Drosophila* mobility, flies were fed with multiple concentrations of rotenone (10, 25, 50, 100, 250, 500, and 1,000 μM) prepared in 5% sucrose and the control flies remain in 5% sucrose using filter disc method. Flies of different age groups showed mobility defects after particular time points. Mobility defects were quantified using a negative geotaxis assay. Under normal conditions, we observed that 90% of the flies could reach the top of the column in 12 s while rotenone-induced flies could not. The higher the concentration of rotenone, the fewer flies could climb. In early health span (Figure 2A) flies, 500 μM concentration of rotenone induces a clear mobility defect on the 5th day but no mortality was observed. In the case of late health span (Figure 2B) and transition phase (Figure 2C) flies, 25 and 10 μM rotenone, respectively, could induce mobility defects on the 2nd day and there was no observable mortality at all these time points. However, the mobility was significantly reduced to 30, 34, and 26% for early health span, late health span, and transition phase flies, respectively. Hence, these concentrations and time points were selected for further assays.

### Rotenone mediated inhibition of mitochondrial complex I: Relevance of the fly model to human Parkinson’s disease

**FIGURE 3:**
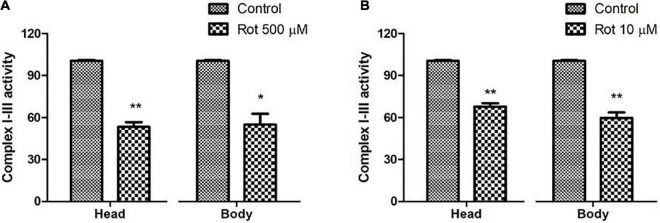
Rotenone inhibits mitochondrial Complex I activity. Upon feeding the early health span fly with 500 μM rotenone for 5 days **(A)** and 10 μM ROT for 2 days during the transition phase **(B)**, there is a significant decrease of ∼45 and ∼35%, respectively, in the complex I–III activity of the respiratory chain in both the head and body parts of *Drosophila melanogaster*. Statistical analysis was performed using a *t*-test (compared to control). *p < 0.05, **p < 0.01.

The mitochondrial complex I–III activity was assayed to explore the inhibition of mitochondrial complex I in rotenone-treated flies. Mitochondrial complex I activity is shown to have been significantly reduced in *substantia nigra*, skeletal muscles, and platelets of PD patients (Bose and Beal, 2016). In the present model, upon rotenone treatment, complex I–III activity was significantly inhibited ranging from 45 ± 4.77% in both the head and body parts of the flies during the health span (Figure 3A); while 35 ± 5.46% reduction was observed during the transition phase (Figure 3B). This reduction in complex I–III activity indicates the impairment of complex I of the electron transport chain.

### Whole-brain immunostaining indicates rotenone does not cause a loss in the number of DAergic neurons but diminishes tyrosine hydroxylase synthesis

**FIGURE 4:**
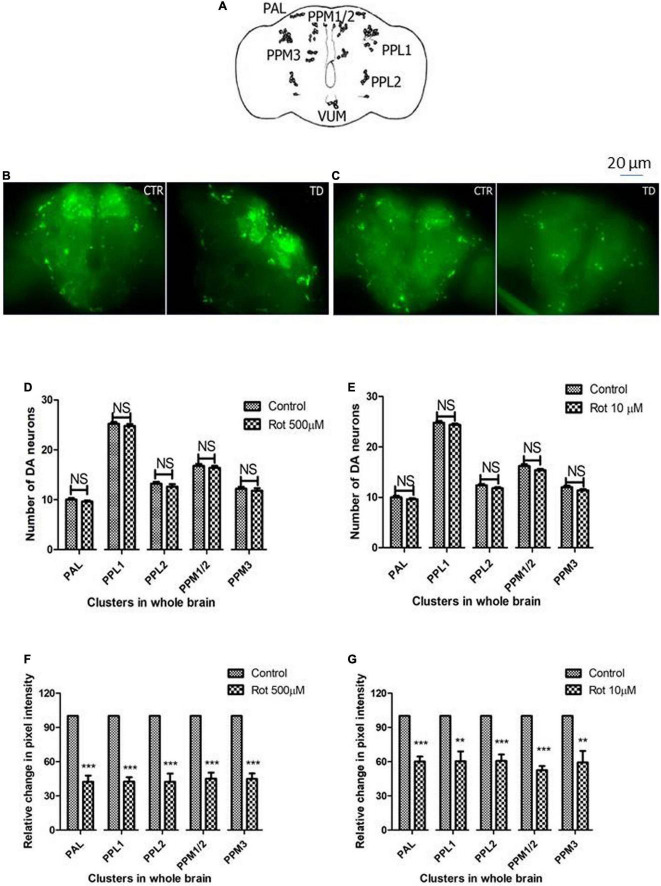
Characterization of DAergic neurodegeneration in the whole fly brain through anti-TH antibody immunostaining reveals that there is no loss in the number of DAergic neurons. However, rotenone leads to “neuronal dysfunction” as characterized by quantification of DAergic neuronal fluorescence intensity that is proportional to the amount of TH protein. Cartoon showing the position of DAergic neurons in the whole brain of *Drosophila melanogaster* **(A)**. Brain image of control and induced PD conditions during early health span **(B)** and transition phase **(C)**. Quantification of DAergic neurons reveals that there is no loss of neuronal number *per se* in both life stages, early health span **(D)**, and transition phase **(E)**, whereas the quantification of fluorescence intensity (of fluorescently labeled secondary antibodies that target primary antibody anti-TH) reveals a significant decrease in TH protein in both life stages, that is, health stage **(F)** and transition stage **(G)**. CTR, Control; TD, Treated with rotenone; PAL, Protocerebral anterior lateral; PPL, Protocerebral posterior lateral; PPM, Protocerebral posterior medial. Statistical analysis was performed using a *t*-test (compared to control). **p < 0.01, ***p < 0.0001, NS, not-significant.

The adult *Drosophila* brain consists of six DAergic neuronal clusters in each brain hemisphere (Figure 4A; Whitworth et al., 2006). To investigate DAergic neuronal dysfunction in rotenone-administered flies, brains were dissected and immunostained for tyrosine hydroxylase (TH) (rate-limiting enzyme in the synthesis of dopamine) in early health (Figure 4B) and transition phase flies (Figure 4C). Upon quantifying the number of DAergic neurons, no significant difference was observed in all the six clusters analyzed in PD brains as compared to control (Figures 4D,E). There are slight variations in the number of neurons even in the control groups, which can be attributed to natural variation. This result is consistent with the earlier comprehensive study performed by Navarro et al. (2014). Then, to understand if there is any variation in the level of synthesis of TH, we quantified the fluorescence intensity of the DAergic neurons (fluorescently labeled secondary antibody targets the primary antibody (anti-TH). Hence, fluorescence intensity is directly proportional to the level of TH protein synthesis). Upon exposure to rotenone, the fluorescence intensity of DAergic neurons of the whole brain mount showed a significant reduction (40–50%) as compared to control groups (Figures 4F,G). This reduction in fluorescence intensity (quantification of fluorescence intensity of GFP reporter), not the loss of neurons *per se*, is termed “neuronal dysfunction” by Navarro et al. (2014). This observation illustrates that DA neuronal structure is not degenerated (hence any loss in the number of neurons), but TH synthesis is diminished.

### Rotenone induces alteration in brain dopamine and its metabolites (3,4-Dihydroxyphenylacetic acid and Homovanilic acid) in both health and transition phases

**FIGURE 5:**
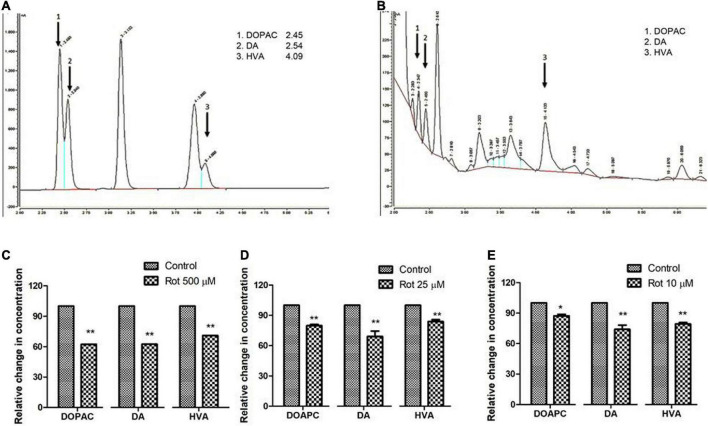
Quantification of DA and its metabolites, HVA, and DOPAC using HPLC in fly brain homogenate: Chromatogram of standard DA, HVA, and DOPAC **(A)** shows specific retention time and chromatogram for fly brain tissue extract **(B)**. The relative level of DA and its metabolites at the selected time points in three stages of the life span, that is, early health span **(C)**; late health span **(D)**; transition phase **(E)** indicates diminished levels of DA, HVA, and DOPAC illustrating altered DA metabolism in brain-specific fashion in the PD model. Statistical analysis was performed using a *t*-test (compared to control). *p < 0.05, **p < 0.001.

To understand the role of rotenone-induced neurotoxicity on DA metabolism, we quantified the level of brain dopamine (DA) and its metabolites (HVA and DOPAC) using HPLC with an electrochemical detector. Analysis reveals that the level of DA is significantly decreased by 38, 28, and 26% under treated conditions in early health span (Figure 5C), late health span (Figure 5D), and transition phase (Figure 5E) flies, respectively. The level of DOPAC is also significantly decreased in early health span flies (38% compared to control), late health span flies (20% as compared to control), and transition phase flies (13% as compared to control) as observed in human PD patients (Andersen et al., 2017) and *Drosophila* PD model exposed to paraquat (Shukla et al., 2016). The level of HVA is significantly reduced (29, 16, and 21% compared to control) during the early health phase, late health phase, and transition phases of the fly life span. These results indicate altered rotenone-induced DA metabolism in the fly brain during early, late health, and transition phases.

### Curcumin rescues the mobility defects induced by rotenone during health phase but not during transition phase

**FIGURE 6:**
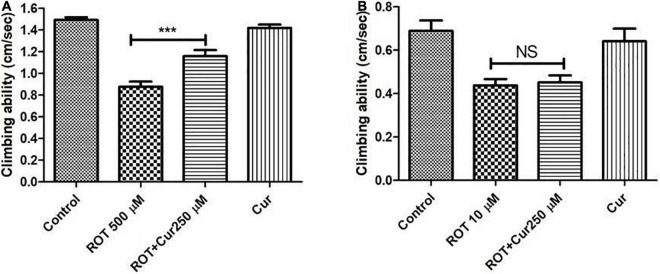
Negative geo-taxis assay of early health phase and transition phase fly in co-feeding regime: Feeding the fly with ROT alone led to a significant reduction in climbing ability which could be significantly improved upon co-feeding with 250 μM curcumin during the early health phase **(A)** but not during the transition phase **(B)** of the fly. Climbing ability was assessed on the 5th day for the early health phase fly, while it was assessed on the 2nd day in case of the transition phase. Feeding the fly with curcumin *per se* did not affect the mobility. One-way ANOVA followed by the Newman–Keuls Multiple Comparison Test showed a significant difference in mobility. ***p < 0.0001; NS, Not significant.

To test the proposed hypothesis, the negative geotaxis ability of the rotenone-mediated adult early health and transition phase fly was assayed to decipher the mobility alterations under the curcumin co-feeding conditions. The distance that the fly climbed in 12 s was assayed after 5 and 2 days in case of the early health phase and transition phase, respectively, after exposure to rotenone or rotenone along with 250 μM curcumin. The rotenone-treated fly exhibited mobility defects. The speed of the fly was significantly improved upon co-feeding with curcumin during the health phase (Figure 6A) but not during the transition phase (Figure 6B) as compared to the fly fed with rotenone alone. Feeding the fly with curcumin alone had no adverse influence on the mobility performance of the fly. This result indicates that the efficacy of genotropic drug/nutraceutical-like curcumin is life phase-specific indicating the importance of employing life stage-specific animal models to screen drugs/nutraceuticals for their DAergic neuroprotective efficacy and to understand pathophysiology of late-onset neurodegenerative diseases such as PD.

## Discussion

The adult life of *Drosophila* was categorized by Arking et al. (2002) into health, transition, and senescent stages. Our laboratory characterized these stages in OK as follows: health span wherein there is no natural death occurs (0–30 days), transition span (slight decline in the mortality curve showing 10% death; 31–60 days), and senescence span (steady decline in mortality curve represented by the window between the end of transition phase till maximum life span of the fly; 61–120 days) (Phom et al., 2014). A review of the literature reveals that from 2004 to date, there are 63 research articles relating to rotenone-mediated fly PD models (source: Pubmed). In all these works, the age of the *Drosophila* employed varies from 3 to 20 days (Coulom and Birman, 2004; Cassar et al., 2015; Pandareesh et al., 2016; Nguyen et al., 2018; Sur et al., 2018; Maitra et al., 2021). Meaning all these animals belong to the adult health phase. But late-onset NDDs such as PD sets in during the transition phase of adult life! It has been shown that gene expression profiles vary among different life stages and the observed variation is similar among flies, mice, and humans (Pletcher et al., 2002). Hence, it is possible that molecular targets of genotropic nutraceuticals/therapeutic agents may not be present in all the life phases leading to the failure of young animal-based therapeutic strategies for late-onset NDDs such as PD (Phom et al., 2014). This critical aspect of the aging process is important to be taken into consideration while studying the pathophysiology of NDD such as PD. So, there is an immediate requirement of developing and characterizing life stage-specific animal models for PD.

Multiple epidemiological studies point toward a strong relationship between environmental toxin exposure and sporadic PD. Results from our present study reveal that *Drosophila* is susceptible to rotenone in a time and dose-dependent manner (Figure 1) and rotenone exposure leads to mobility defects (Figure 2). The primary objective of the present study is to develop an adult life stage-specific *Drosophila* model to understand the pathophysiology associated with late-onset NDD such as PD. The present study shows that the rotenone concentration of 1,000 μM is toxic to early health span flies, while 50 μM and above concentrations are highly toxic in the case of late health span and transition phase flies. While developing animal models of DA degeneration/PD, it is important to avoid acute concentrations of neurotoxicants. Acute concentrations lead to a systemic failure of an organism. Hence, observations made in such models may not throw light into the degeneration process of a specific set of neurons such as DAergic in the case of PD.

We aim to decipher the pathophysiology related to PD well before the occurrence of death and at the point of exhibiting disease phenotypes such as mobility defects. Hence, we chose 500 μM rotenone for 5 days in case of early health span flies, 25 μM rotenone for 2 days in case of late health span flies, and 10 μM rotenone for 2 days for transition phase flies [all these concentrations and duration of exposures are carefully picked up after performing comprehensive longevity studies (Figure 1) and characterizing mobility defects (Figure 2)] in such a way that though fly exhibits mobility defects at these points, mortality due to systemic failure occurs much later.

It has been demonstrated that rotenone-mediated DAergic neurodegeneration is due to the inhibition of mitochondrial complex I activity and subsequent failure of the energy-generating mechanism of the cells (Coulom and Birman, 2004; Innos and Hickey, 2021). Inhibited mitochondrial complex activities thereby causing oxidative damage to DAergic neurons have been demonstrated in PD patients (Zilocchi et al., 2018; Carling et al., 2020). Our result shows the reduced activity of complex I–III indicating the inhibition of complex I of the ETC (Figure 3). The level of inhibition is lower in aged flies as seen in aged mice (Genova et al., 1997). This could be due to a significant decrease in sensitivity of NADH oxidation in old aged animals, as the rotenone binding site encompasses a complex I subunit encoded by mt-DNA which is on the verge of cellular senescence (Genova et al., 1997). Inhibition of complex I disrupt electron flow thereby decreasing ATP production and increasing ROS production (Mizuno et al., 1987) leading to the death of the DAergic neurons. All these studies validate the present rotenone-mediated fly PD model.

Upon analyzing the DAergic neurons, we found that the number of DAergic neurons remain unchanged in the PD model as compared to the control (Figure 4). Our results are in agreement with the findings from other labs (Meulener et al., 2005; Pesah et al., 2005; Navarro et al., 2014). Loss of DA neuronal number has been an issue of controversy in fly models of PD (Navarro et al., 2014). By quantifying the nuclear GFP (green fluorescent protein) reporter (driven by TH-gal4), Navarro et al. (2014) re-evaluated and clarified the issue that in both genetic and toxin-based *Drosophila* models of PD, though there is no loss of DA neurons, the level of TH protein synthesis is diminished (“neuronal dysfunction”). We quantified the fluorescence of fluorescently labeled secondary antibody which is targeted against the primary antibody anti-TH. Results indicate that the fluorescence intensity is significantly reduced to 40–50% under induced PD conditions, which suggests the reduced levels of TH and the DAergic “neuronal dysfunction” (Navarro et al., 2014). Therefore, our study illustrates that in the present model, though there is no loss of DA neurons *per se*, there is a significant reduction in the TH synthesis. Hence, it is possible that DA synthesis is diminished which is further characterized using HPLC.

The oxidation of dopamine into its metabolites is a normal phenomenon to keep dopaminergic neurons intact in the substantia nigra (Segura-Aguilar et al., 2014). The effects of aldehyde dehydrogenases in three different life stages of mice [young (5–8 months); middle-aged (12–14 months) and old (18–27 months)] suggested that deletion of two isoforms of aldehyde dehydrogenase resulted in Parkinsonism-like age-related reductions in monoamines and their metabolites and degeneration of dopaminergic neurons (Wey et al., 2012). Our laboratory has previously shown a significant reduction in the level of dopamine under paraquat-induced PD conditions (Phom et al., 2014). The present study also showed a significant reduction in the level of DA (*p* < 0.001) in all the life stages (Figure 5). This result validates the previous observation of diminished levels of TH as determined through quantification of fluorescence intensity of fluorescently labeled secondary antibodies (Figure 4). The level of DOPAC which is downstream of DA is significantly reduced in early health span (*p* < 0.001), late health span (*p* < 0.01), and transition phase (*p* < 0.01) flies. The level of HVA is significantly reduced during the health (*p* < 0.001) and transition phases (*p* < 0.01) of the fly life stages. There has been no study so far on the DA and its metabolites in three important stages of the life span of flies. We report here for the first time the effect of rotenone on DA metabolism-related neurodegeneration in different life stages of *Drosophila*. During health and transition phases upon rotenone treatment, levels of DA significantly diminished (38 and 26%, respectively). However, DA catabolism into HVA and DOPAC in the transition phase brain is higher compared to the levels in the health span brain (HVA and DOPAC levels are close to the control brains) suggesting the influence of rotenone on brain DA metabolism.

From the present study, we summarize that exposure of *Drosophila* to rotenone demonstrates characteristic features of PD: concentration-dependent lethality, locomotor deficits, complex I inhibition, DAergic neuronal dysfunction, and depletion in dopamine levels.

To test our hypothesis, in the present life phase-specific rotenone-mediated fly model of PD, we screened curcumin (a nutraceutical/drug that has been employed in about 438 clinical trials (source: ClinicalTrials.gov) for multiple human disease conditions) for its efficacy. Results illustrate that curcumin rescues rotenone-mediated mobility defects only during the adult health phase, but not during transition phase (Figure 6). This suggests that the efficacy of genotropic drug/nutraceutical-like curcumin is life phase-specific; emphasizing the necessity of employing life stage-specific animal models to screen drugs/nutraceuticals for their DAergic neuroprotective efficacy and to understand the pathophysiology of late-onset neurodegenerative diseases such as PD. We have comprehensive insights and data relating to curcumin’s life phase-specific neuroprotective efficacy. This understanding will be critical in formulating healing strategies for PD.

Life phase-specific models are crucial to decipher underlying molecular mechanism(s) of neuroprotection/degeneration; knowledge of which is essential in developing novel therapeutic strategies for PD.

## Data availability statement

The original contributions presented in this study are included in the article/Supplementary material, further inquiries can be directed to the corresponding author.

## Author contributions

MA: performing the experiments, data acquisition, analysis of the data, and drafting the manuscript. LP, ZK, PM, AD, RC, AT, NJ, and KN: performing experiments. SY: conception, design of the study, interpretation of the data, and manuscript preparation. All authors contributed to the article and approved the submitted version.
