# Supplementary material for: Adult health and transition stage-specific rotenone-mediated Drosophila model of Parkinson’s disease: Impact on late-onset neurodegenerative disease models
Source: Front Mol Neurosci. 2022 Aug 9;15:896183. doi: 10.3389/fnmol.2022.896183 (PMC9398202; doi:10.3389/fnmol.2022.896183)
Supplement: Supplementary file 1 [file Data_Sheet_1.PDF]

Parameter
Table Analyzed 30day flies@2day

One-way analysis of variance
P value P<0.0001
P value summary ***
Are means signif. different? (P < 0.05) Yes
Number of groups 8
F 61.25
R squared 0.8562

Bartlett's test for equal variances
Bartlett's statistic (corrected) 6.666
P value 0.4645
P value summary ns
Do the variances differ signif. (P < 0.05) No

ANOVA Table	SS	df	MS
Treatment (between columns)	4.194	7	0.5991
Residual (within columns)	0.7042	72	0.009781
Total	4.898	79	

Newman-Keuls Multiple Comparison Test	Mean Diff.	q	Significant? P < 0.05?	Summary
Rot 1000mM vs Control	-0.7139	22.83	Yes	***
Rot 1000mM vs Rot 10mM	-0.6611	21.14	Yes	***
Rot 1000mM vs Rot 25mM	-0.3778	12.08	Yes	***
Rot 1000mM vs Rot 50mM	-0.3194	10.21	Yes	***
Rot 1000mM vs Rot 100mM	-0.2556	8.171	Yes	***
Rot 1000mM vs Rot 250mM	-0.2139	6.839	Yes	***
Rot 1000mM vs Rot 500mM	-0.1500	4.796	Yes	**
Rot 500mM vs Control	-0.5639	18.03	Yes	***
Rot 500mM vs Rot 10mM	-0.5111	16.34	Yes	***
Rot 500mM vs Rot 25mM	-0.2278	7.283	Yes	***
Rot 500mM vs Rot 50mM	-0.1694	5.418	Yes	**
Rot 500mM vs Rot 100mM	-0.1056	3.375	No	ns
Rot 500mM vs Rot 250mM	-0.06389	---	No	ns
Rot 250mM vs Control	-0.5000	15.99	Yes	***
Rot 250mM vs Rot 10mM	-0.4472	14.30	Yes	***
Rot 250mM vs Rot 25mM	-0.1639	5.240	Yes	**
Rot 250mM vs Rot 50mM	-0.1056	3.375	No	ns
Rot 250mM vs Rot 100mM	-0.04167	---	No	ns
Rot 100mM vs Control	-0.4583	14.66	Yes	***
Rot 100mM vs Rot 10mM	-0.4056	12.97	Yes	***
Rot 100mM vs Rot 25mM	-0.1222	3.908	Yes	*
Rot 100mM vs Rot 50mM	-0.06389	---	No	ns
Rot 50mM vs Control	-0.3944	12.61	Yes	***
Rot 50mM vs Rot 10mM	-0.3417	10.92	Yes	***
Rot 50mM vs Rot 25mM	-0.05833	1.865	No	ns
Rot 25mM vs Control	-0.3361	10.75	Yes	***

Rot 25mM vs Rot 10mM	-0.2833	9.059	Yes	***
Rot 10mM vs Control	-0.05278	1.688	No	ns
